# Brain in Relation to Mind

**Published:** 1900-12

**Authors:** 


BRAIN IN RELATION TO MIND. By J. SANDERSON CHRISTISON, M. D., Author of "Crime and Criminals," etc. Duodecimo, pp. 142. Second edition. Chicago: The Meng Publishing Co. 1900. [Price, $1.25.]

This brochure by Dr. Christison is addressed to physicians as well as laymen and cites facts rather than to present arguments and aims to establish these facts by the best of references. The author gives the views of Dr. Hughlings Jackson on the Relation of brain to mind in chapter first, and asserts that notwithstanding the correctness of Dr. Jackson's statement that the materialistic doctrine of mind has been destroyed, it seems to be reviving with more assertiveness than ever. In chapter II. the author takes up the consideration of brain cells and their relation, and in chapter III. the theory of sensory and motor centers, condensing in a few pages the care of our present state of knowledge regarding these anatomical elements.

In chapter IV. the theory of mind localisation is discussed, the author citing many cases where injury to the frontal lobes has not impaired materially the mental faculties and concludes that the brain possesses functional substitution, a capacity that could not exist if brain and mind were identical.

Chapter V. is devoted to brain fever in relation to mind and deals mostly with the comparative anatomy of the brain.

Chapter VI., the size of the brain is considered, and it is found that the size bears no fixed relationship to any one psychic quality or combination of such qualities. In the last chapter the author discusses the normal mind or the prompt and coördinate action of all the mental faculties coëxisting with pacific disposition or temper. The different mental states, the faculties of the mind and normal decadence are clearly discussed and rigidly interpreted. The work is readable to layman and professional and is a very succinct psychological study of the most complex subject of the day.

The paper, print and binding are of good quality.

